# Is dietary quality associated with depression? An analysis of the Australian Longitudinal Study on Women’s Health data

**DOI:** 10.1017/S0007114522002410

**Published:** 2023-04-28

**Authors:** Megan Lee, Joanne Bradbury, Jacqui Yoxall, Sally Sargeant

**Affiliations:** 1 Bond University, Gold Coast Campus, Robina, Australia; 2 Southern Cross University, Gold Coast Campus, Coolangatta, Australia; 3 Southern Cross University, Lismore Campus, Lismore, Australia

**Keywords:** Dietary patterns, Depression, Depressive symptoms, Australian Longitudinal Study of Women’s Health, Diet quality

## Abstract

Depression is a chronic and complex condition experienced by over 300 million people worldwide. While research on the impact of nutrition on chronic physical illness is well documented, there is growing interest in the role of dietary patterns for those experiencing symptoms of depression. This study aims to examine the association of diet quality (Dietary Questionnaire for Epidemiological Studies version 2) and depressive symptoms (Centre for Epidemiological Studies for Depression short form) of young Australian women over 6 years at two time points, 2003 (*n* 9081, Mean age = 27·6) and 2009 (*n* 8199, Mean age = 33·7) using secondary data from the Australian Longitudinal Study on Women’s Health. A linear mixed-effects model found a small and significant inverse association of diet quality on depressive symptoms (*β* = −0·03, 95 % CI (−0·04, −0·02)) after adjusting for covarying factors such as BMI, social functioning, alcohol and smoking status. These findings suggest that the continuation of a healthy dietary pattern may be protective of depressive symptoms. Caution should be applied in interpreting these findings due to the small effect sizes. More longitudinal studies are needed to assess temporal relationships between dietary quality and depression.

Over the last decade, there has been an increase in interest in the relationship between nutrition and mental health in epidemiological studies^(1–3)^. Poor diet and poor mental health are leading causes of global mortality and morbidity^(4)^. Mental health disorders currently represent one of the most substantial global burdens of disease, estimated as costing USA$2·5 trillion, including costs such as medication, psychotherapy, workplace absenteeism and income losses^(4–7)^. In Australia between 2017 and 2018, over 2·5 million people experienced depression, with a prevalence of 10·4 %. Females aged 25 to 34 (11·8 %) reported higher rates than their male counterparts (10·2 %)^(8)^. The role of nutrition in chronic lifestyle diseases such as type 2 diabetes^(9)^, CVD^(10–12)^, some cancers^(13,14)^, metabolic syndrome and obesity^(15,16)^ is generally well documented. However, the role of nutrition in mental health is less well known^(17,18)^ and has provoked growing interest in the association between dietary patterns, diet quality and the association with symptoms of depression^(19–22)^.

Research proposes that a wide variety of biological mechanisms are involved in the heterogenous and complex relationship between nutrition and depression including decreased monoamine function, dysfunctional hypothalamic pituitary adrenal axis, neuro-progression/brain plasticity, mitochondrial disturbances^(23,24)^, cytokine-mediated inflammatory processes, increased oxidative stress, immune responses^(25)^, immuno-inflammation, gut dysbiosis and gut/brain axis relationships^(24,26)^. However research on the role of these biological mechanisms and nutrition in depression is relatively new and focuses on single food components^(27–29)^ and nutritional supplementation rather than whole-of-diet sources^(17,30–32)^.

Dietary patterns are defined as ‘the quantity, variety or combination of different foods and beverages in a diet and the frequency with which they are habitually consumed’ (Sanchez-Villegas *et al*., 2018, p. 4). Diet quality is defined as ‘The nutritional adequacy of an individual’s dietary pattern and how closely this aligns with national dietary guidelines’^(33)^ and is commonly used as a measure of healthy and unhealthy dietary patterns assessing high and low diet quality^(22,34,35)^. Healthy dietary patterns are generally rich in fresh vegetables and fruits, nuts, seeds, whole grains, fermented foods, legumes and water^(36)^. Most of the research on dietary patterns and depression involves observational epidemiological studies that indicate an association between healthy dietary patterns and decreased depressive symptoms^(37,38)^, while unhealthy dietary patterns high in ultra-processed, refined and sugary foods are associated with higher symptoms of depression^(39,40)^. Currently, there are four randomised control trials that have assessed the effect of changing from an unhealthy to a healthy dietary pattern^(41–44)^. All four Australian randomised control trials found a significant improvement in depression scores between the intervention and social control groups. However, evidence arising from meta-analyses and systematic reviews shows inconsistent or inconclusive findings when the research is viewed as a whole^(39,40,45–49)^. These findings could be clarified through further prospective longitudinal studies on dietary intake and depression^(50)^.

One study that examines both diet quality and depressive symptoms prospectively is the Australian Longitudinal Study of Women’s Health (ALSWH^(51)^). Previous research using ALSWH data has examined the role of diet quality and depressive symptoms using longitudinal analysis^(27,52–55)^. Not all have found an association. For instance, Lai *et al.* ^(53)^ utilised data from the ALWSH, which focused on Australian women born between 1946 and 1951 and found a significant inverse association between diet quality and depressive symptoms (*β* = − 0·24, *P* = .001). However, they also demonstrated that these associations were no longer significant after adjusting for covarying factors such as BMI, smoking and alcohol status and physical activity (*β* = − 0·04, *P* = .100). This result suggested that a relationship between diet and depression may be explained by covarying lifestyle factors^(56–59)^. A follow-up analysis using the ALSWH data by the same authors^(52)^ resulted in lower odds of depressive symptoms in high (OR = 0·86; 95 % CI (0·77, 0·96)) and moderate (OR = 0·94; 95 % CI (0·80, 0·99)) diet quality tertiles compared with low diet quality. This suggests that maintaining diet quality over the long term could reduce the odds of depressive symptoms. The authors recommended more longitudinal research using a younger cohort from the ALSWH.

One cross-sectional survey measured diet quality and depressive symptoms in 3963 Japanese middle-aged women (M = 47·9, SD = 4·2 years)^(60)^. After adjusting for covariates, they found that high diet quality was associated with lower depressive symptoms compared with participants with low diet quality (OR = 0·65, 95 % CI (0·45, 0·78)). Apart from this study, there is a paucity of research that focuses on the relationship between diet quality and depressive symptoms in young women. To fill this gap in the literature, we conducted a secondary analysis of the ALSWH data examining whether there is a longitudinal association between dietary quality and depressive symptoms in a cohort of young Australian women. The sample for the current study was sourced from the 1973–1978 cohort, including data from two time points in 2003 (Mean age = 27·6, sd = 1·5) and 2009 (Mean age = 33·7, sd = 1·5) where diet quality, depressive symptoms and all covarying factors were measured. These data have not been analysed in previous research, and it is our aim to replicate the previous studies analysis with fresh data.

## Methods

### Participants

The ALSWH^(51)^ is a continuing longitudinal cohort study of more than 50 000 women in Australia. It is divided into four age cohorts of women born between 1921 to 1926, 1946 to 1951, 1973 to 1978 and 1989 to 1995^(53,61)^. On commencement in 1996, 40 392 women were recruited into the first three cohorts, followed by 17 069 into the 2012 fourth cohort^(62)^. Participants were randomly selected from the Australian health insurance database, Medicare, including all Australian permanent residents. Response rates for each cohort were estimated as 37 % to 40 % (1921–1926), 53 % to 56 % (1946–1951), 41 % to 42 % (1973–1978) and 70 % (1989–1995). Women completed a survey containing questions relating to their health outcomes every 3 to 4 years from 1996 to 2018. The study protocol followed the Declaration of Helsinki guidelines^(63)^, and formal ethical approval was given by the Human Research Ethics Committees of the University of Queensland and the University of Newcastle in Australia. Participants supplied informed consent before being included^(51)^.

The analysis for this particular paper is targeted to the cohort of women who were born between 1973 and 1978. Participants completed a baseline questionnaire in 1996 (*n* 14 247) and every 3 years thereafter; 2000 (*n* 9688), 2003 (*n* 9081), 2006 (*n* 9145), 2009 (*n* 8199), 2012 (*n* 8009), 2015 (*n* 7186) and 2018 (*n* 7121). The sample for the current study includes data from two time points in 2003 (*n* 9081, Mean age = 27·6) and 2009 (*n* 8199, Mean age = 33·7) where diet quality, depressive symptoms and all covarying factors were measured.

### Materials

#### Depressive symptoms ()

The Centre for Epidemiological Studies Depression short form (CESD-10) was used in the ALSWH to ‘assess depressive symptoms during the past week at each survey’^(64)^. The CESD-10 includes ten of twenty items from the original CESD^(65)^. Response format is a four-point Likert scale, ranging from 0 (none of the time) to 3 (all of the time). Total scores are obtained by summing across items ranging from 0 to 30, with higher scores indicating greater depressive symptom severity. The CESD-10 was designed to measure depressive symptoms experienced in the general population rather than provide a clinical diagnosis. A score greater than ten is the standard cut-off to classify people experiencing depressive symptoms^(65)^.

The CESD-10 has high internal consistency (Cronbach *α* = 0·88), 92 % specificity identifying those without depressive symptoms, 91 % sensitivity identifying those with depressive symptoms and 92 % positive predictive values reflecting the presence of depressive symptoms^(66)^.

#### Food frequency questionnaire

The Dietary Questionnaire for Epidemiological Studies version 2 (DQES v2) was administered to the selected cohort in 2003 and again in 2009 in the ALSWH. The DQES v2 is a self-report FFQ developed by the Cancer Council Victoria that measures dietary intake in epidemiological studies^(67)^. In the DQES v2, participants report dietary consumption of seventy-two foods over the previous 12 months. Additional questions are asked on the frequency of consuming fruit, vegetables, meat, meat alternatives, milk, bread, butter, spreads, cheese, sugar and eggs^(67)^. A study of 237 Australian participants indicated test-retest reliability of the DQESv2 with weighted *κ* of 0·58 over 12 months^(68)^. This test-retest reliability is similar to the widely used Commonwealth Scientific and Industrial Research Organisation FFG^(69)^. The DQES v2 has been used in previous research relating to dietary patterns and depression in women^(52,53)^ and for most food types was comparable to other FFQ (Hodge *et al*., 2000). As a measure of diet quality, the Australian Recommended Food Score (ARFS) was applied to data collected using the DQES v2^(70)^.

#### Diet quality (Australian Recommended Food Score)

The ARFS uses scoring in line with the Australian Dietary Guidelines (ADG) and the Australian Guide to Eating^(71)^. The ARFS is calculated by summing points within eight subscales: vegetable intake (twenty-one items), fruit (twelve items), protein foods (seven items), plant-based protein (six items), bread and cereals (thirteen items), dairy products (eleven items), water (one item) and fats (two items). Foods are given one point for a frequency of more than once/week. Scores range from 0 to 73, with higher values corresponding to healthier dietary quality. The ARFS has been validated using the Australian Eating Survey^(33)^ and used in previous studies using ALSWH cohorts^(52,53)^.

#### Covariates

Covarying factors commonly associated with depressive symptoms in the literature were included BMI – measured by calculating self-report weight in kilograms divided by height in metres squared; social functioning – measured by averaging two items of the thirty-six-Item Short Form Survey (SF-36^(72)^); with *α* reliability levels of 0·85^(73)^; anxiety – measured using self-report of clinical diagnosis (yes/no); alcohol status – measured using three self-report items (independent of the ARFS diet quality score) on how often and how much alcohol was consumed each week and engagement in binge drinking (no risk; binge less than once a month; binge once a month or more; more than two drinks/d on average) in line with classifications from the National Health and Medical Research Council^(74)^; smoking status – measured using three self-report items on how often and how many cigarettes smoked each week and; education level – no qualification, school certificate, higher school certificate, trade certificate, diploma, undergraduate degree and postgraduate degree.

#### Data analysis strategy

We calculated descriptive statistics for both time points (2003 and 2009) using mean, median, standard deviation, histograms and boxplots for continuous variables (CESD-10, ARFS, SF36 and age) and frequencies, percentages and bar charts for categorical variables (BMI, clinically diagnosed depression and anxiety, education, marital, smoking and alcohol status). Assumptions for linear mixed-effects model, including linearity and equal variance, were assessed using histograms and scatterplots of residuals^(75)^. Normality of distributed errors was observed using probability-probability (pp) and quantile-quantile (qq) plots. The Akaike Information Criterion (AIC) and log-ratio tests were used to assess the model fit. AIC is used to determine the information lost by adding a variable to the model, with lower AIC indicating a better fit^(76)^. Criteria for retaining or excluding variables in the final models were a substantial reduction of the AIC and a significant log-ratio test. Only data that were complete for both time points 2003 and 2009 for each participant were included in the model (*n* 8199).

A linear mixed-effects model was used to predict depression total scores (continuous CESD-10 score) as a function of diet quality (continuous ARFS total score) and year (2003 and 2009), with participant as a random effect. The model was formulated as follows with _i_ indicating individual and _j_ indicating time:

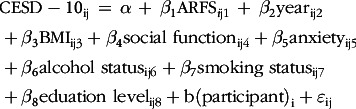




Continuous covariates (BMI and social function) and categorical covariates (anxiety, alcohol status, smoking status, physical activity, geographical location, marital status, socio-economic status and education level) were added one at a time in a stepwise fashion. Each step in the model reduced the AIC and was associated with a significant log-ratio test, apart from physical activity, geographical location, socio-economic status and marital status. Therefore, these four variables were removed. The final model was specified with the CESD-10 as the outcome, ARFS total score as the predictor, with covariates as BMI, social function, anxiety, alcohol status, smoking status and education level added stepwise.

## Results

### Participant characteristics

Participant characteristics of women at 2003 and 2009 are summarised in Table 1. At baseline, in 2003, 9081 participants were included (mean age = 27·6, sd = 1·5). At the final time point in 2009, 8199 (90 %) participants remained (mean age = 33·7, sd = 1·5). At this time point, 77 % of women were partnered, 56 % had completed a university degree, 41 % had smoked cigarettes in their lifetime, 88 % currently consumed alcohol and 10 % were clinically diagnosed with anxiety. According to WHO^(77)^ BMI categorisation, 45 % of women were classified as overweight or obese compared with 37 % in 2003. In 2003, sample mean diet quality was 29·2 (sd = 9·3) compared with 33·2 (sd = 9·3) in 2009. In relation to depression, 13 % (2003) and 18 % (2009) of the cohorts were clinically diagnosed with depression, while CESD-10 scores across the cohort fell below the cut-off for experiencing depressive symptoms 7·0 (sd = 5·3) in 2003 and 6·4 (sd = 5·2) in 2009.

**Table 1.** Participant characteristics over time (Numbers and percentages)

	2003 (*n* 9081)		2009 (*n* 8199)	
	*n*	%	*n*	%
Education level				
No qualification	89	1	54	1
High school	2208	25	1438	18
Trade/certificate	2158	24	2040	26
University degree	4372	50	4565	56
Total	8827		8097	
Marital status				
Partnered	5549	61	6320	77
Unpartnered	3496	39	1849	23
Total	9045		8169	
Smoking status				
Never smoked	5171	57	4882	60
Ex-smoker	1674	19	2105	26
Smokes <10 d	1102	12	548	7
Smokes 10–19 d	713	8	406	5
Smokes > 20 d	388	4	240	3
Total	9048		8181	
Alcohol status				
Non-drinker	731	8	990	12
Low-risk drinker	5522	61	4838	59
Rarely drinks	2462	27	1977	24
Risky drinker	280	3	287	4
High-risk drinker	49	1	74	1
Total	9044		8166	
BMI				
Underweight	363	4	209	3
Normal weight	4732	58	4178	52
Overweight	1792	22	2053	25
Obese	1217	15	1614	20
Total	8104		8054	
Diagnosed depression				
Yes	1125	13	1339	18
No	7810	87	6229	82
Total	8935		7568	
Diagnosed anxiety				
Yes	545	6	753	10
No	8390	94	6815	90
Total	8935		7568	
	Mean	sd	Mean	sd
Age (years)	27·6	1·5	33·7	1·5
CESD-10	7·0	5·3	6·4	5·2
SF36 SF	80·2	22·5	82·7	22·2
ARFS	29·2	9·3	33·2	9·3

CESD-10, centre for epidemiological studies depression score; SF36 SF, medical outcomes short-form – social function score; ARFS, Australian recommended food score.

### Linear mixed-effects model

In the unadjusted model, there was a small, significant inverse association of ARFS on CESD-10 (*β* = -0·06, *P* < .001), indicating that for every point increase in diet quality, as measured by the ARFS total score, there was a 0·06-point reduction in depressive symptoms, as measured by the CESD-10 total score (online supplementary material). Each step in the model reduced the AIC and was associated with a significant log-ratio test indicating all variables in the table contributed to the model. There was no significant interaction between ARFS total score and year. Therefore, only the main effects were included in the adjusted model. After adjusting for all covariates in the model, there remained a small but significant inverse association of ARFS on CESD-10 (*β* = -0·03, 95 % CI (-0·04, -0·02)), indicating that for each point increase in diet quality there is a .03 point reduction in depressive symptoms (Table 2).

**Table 2.** Adjusted model of ARFS total score on CESD-10 between 2003 (*n* 9081) and 2009 (*n* 8199) (Standardised and unstandardised *β* coefficients)

	*β*	*β*	se	*Z*	*P*	95 % CI
ARFS total score	-0·03	-0·06	0·004	-8·29	< 0·001	-0·04, −0·02
Years						
2003						
2009	–0·21	–0·02	0·064	–3·35	0·001	–0·34, −0·09
Anxiety						
Yes						
No	1·81	0·09	0·128	14·15	< 0·001	1·56, 2·06
BMI	0·08	0·09	0·006	12·20	< 0·001	0·07, 0·09
Social function	–0·13	–0·54	0·002	–81·16	< 0·001	–0·13, −0·12
Alcohol status						
Non-drinker						
Low-risk drinker	–0·04	–0·01	0·122	–0·29	0·772	–0·27, 0·20
Rarely drinks	0·24	0·02	0·130	1·82	0·069	–0·02, 0·49
Risky drinker	0·67	0·02	0·217	3·07	0·002	0·24, 1·09
High-risk drinker	1·33	0·02	0·429	3·09	0·002	0·49, 2·17
Smoking status						
Never smoked						
Ex-smoker	0·26	0·02	0·091	2·84	0·005	0·08, 0·43
Smokes <10 d	0·31	0·02	0·122	2·55	0·011	0·07, 0·55
Smokes 10–19 d	0·80	0·04	0·149	5·36	<0·001	0·51, 1·09
Smokes >= 20 d	0·98	0·03	0·194	5·04	<0·001	0·60, 1·36
Education level						
No qualification						
Year 10	–0·49	–0·02	0·376	–1·31	0·189	–1·23, 0·24
Year 12	–0·57	–0·04	0·366	–1·57	0·116	–1·29, 0·14
Trade certificate	–0·72	–0·02	0·411	–1·76	0·078	–1·53, 0·08
Diploma	–0·65	–0·05	0·363	–1·80	0·073	–1·36, 0·060
Undergraduate	–0·95	–0·09	0·362	–2·61	0·009	–1·66, −0·24
Postgraduate	–0·89	–0·06	0·370	–2·40	0·016	–1·61, −1·63

CESD-10, centre for epidemiological studies depression score; ARFS, Australian recommended food score; *β*, unstandardised *β* coefficient; *β*, standardised *β* coefficient.

## Discussion

This analysis of the ALSWH longitudinal cohort study measured the size and significance of the association between Australian womens’ diet quality and depressive symptoms over 6 years between 2003 and 2009. In a linear mixed-effects model, ARFS scoring was applied to the DQESv2 FFQ to measure diet quality and depressive symptoms between at both time points. After adjusting for covariates, diet quality was inversely associated with depressive symptoms at both time points in this large cohort.

This longitudinal data analysis suggests that a continuation of healthy diet quality predicts lower depressive symptoms for women who already have a healthy diet. The findings are statistically significant after adjusting for various cofactors but had small effect sizes. Caution must be applied when interpreting these results. Although statistically significant, the small effect sizes may not suggest clinical significance. Therefore, it is unclear how much change from an unhealthy to a healthy diet would be needed to infer a result in depressive symptoms in clinical application. A reason for the small effect sizes may be that although 13 % to 18 % of the cohort were clinically diagnosed with depression, overall, when measuring depressive symptoms using the CESD-10 scores, the cohort, on average reported lower than the cut-off scores for depressive symptoms. These findings are comparable with other data analyses using the ALSWH to examine diet quality and depressive symptoms using the same diet quality score (ARFS) and depressive symptoms score (CESD-10) as our study. In their study using the 1946 to 1951 cohort (*n* 7877) of women in the ALSWH who were 67 years old in 2018, Lai *et al*. (2017) reported 6 % reduced odds of depressive symptoms in women who had moderate to high diet quality compared with those who had lower diet quality using the ARFS (moderate *v*. low: OR = 0·94, 95 % CI (0·80, 0·99)), high *v*. low: OR = 0·86, 95 % CI (0·77, 0·96)). Similarly, Rienks *et al*.^(54)^ found that after adjusting for covariates in the 1946 to 1951 cohort, women who had a greater consumption of foods within a Mediterranean dietary pattern had 8 % lower odds of depressive symptoms in 2001 (OR = 0·82, 95 % CI (0·77, 0·88)) and lower odds of depressive symptoms in 2004 (OR = 0·83, 95 % CI (0·75, 0·91)).

Similarly, another longitudinal study measured the association between dietary patterns and depressive symptoms using reduced rank regression in 903 Japanese participants after a 3-year follow-up^(78)^. They found that high adherence compared with low adherence to a healthy Japanese dietary pattern – high in fish, soya products, green tea, vegetables, mushrooms and seaweed was associated with a reduced odds of depressive symptoms (OR = 0·57, 95 % CI (0·35, 0·93)). However, a longitudinal study in the UK assessing dietary patterns and depressive symptoms in young female parents aged 29 to 40 years (*n* 7698) over 4 years^(79)^ found no significant association after adjusting for covariates.

The ARFS measurement of diet quality used in this study implies that the diversity of healthy foods may be an important factor for depressive symptoms. Participants who recorded limited intake from each food group received lower scores on the ARFS than those who recorded a diverse range of different foods, despite eating a large quantity of one type of healthful food^(33)^. This finding suggests that consumption of a broader range of fruits, vegetables, seafood, meats, nuts, seeds, legumes, whole grains and dairy products is as (if not more) important as eating the recommended amount from each food group^(80)^. The components of these foods, such as antioxidants^(81)^, probiotics, prebiotics^(82)^ and complex carbohydrates^(83)^, are known to reduce oxidative stress, chronic inflammation and improve the health of the gut microbiome, which is already identified as contributing to a reduction in depressive symptoms^(84)^. A recent cohort study comparing microbiome samples from 10 000 citizen-scientists from Australia, the UK and the USA found that consuming more than thirty different plant types each week was beneficial to the gut and psychiatric health^(85)^.

This current analysis also found several other predictors of depressive symptoms within the models in addition to diet quality. When assessing diet quality using the ARFS, higher scores in anxiety and BMI were associated with increased depressive symptoms, and women who had higher social functioning had lower odds of depressive symptoms.

This study’s strengths are that the data were collected from a large sample of women representing the Australian population over 6 years. A further strength of this study is the ability to adjust across various socio-demographic and health-related factors within the model enhance the strength of this particular analysis. Furthermore, the ability to assess the impact of diet and socio-demographic factors specific to a female cohort is appropriate as women have higher reported rates of depressive symptoms than men in Australia^(86)^. However, caution must be applied in suggesting a causal role between diet and depression and from the small effects found as the clinical significance of these findings could be uncertain. Clinical significance is distinctly different from statistical significance and indicates whether the association could make a demonstrated, clinically meaningful difference to an individual receiving treatment in the real world^(87)^. Additionally, some variables included in the model are along the causal pathway between depressive symptoms and diet quality (for example, BMI). It was beyond the scope of this cross-sectional study to explore the potential causal roles of variables. Future research could explore mediation and moderation impacts of the other significant variables in the model. A further limitation is that the ARFS measurement of diet quality gave scores for some food types, which were not representative of the definition the authors use of a healthy dietary pattern (high intake of fruits, vegetables, nuts, seeds, legumes, wholegrains, water and low intake of processed, sugary and refined foods) including ice cream, white bread and rice and processed meat products. The ARFS also disadvantaged participants who followed a plant-based dietary pattern as scores were given for meats, eggs and dairy, which are frequently excluded by those following vegetarian and vegan diets. This disadvantage could result in participants who followed a plant-based dietary pattern having reduced scores and potentially being categorised as consuming an unhealthy diet when the opposite may have occurred. Further, the 1-year recall of food consumed was the basis for the diet quality measurement. The reliability of an individual’s recall of foods eaten over this time is questionable and may influence the accuracy of the results^(88)^.

In this report, a longitudinal analysis using linear mixed-effects models, diet quality measured by a FFQ in 2003 and 2009 had a small and statistically significant association. However, this association may not be clinically meaningful. Other predictors of depression were important, including anxiety, BMI and social functioning. This ALSWH longitudinal cohort study analysis has highlighted small inverse findings in the association between dietary patterns and depressive symptoms in Australian women. Further analysis of longitudinal and intervention studies is needed to assess temporal relationships and causality between dietary patterns and depression.

## Supplementary material

For supplementary material accompanying this paper visit https://doi.org/10.1017/S0007114522002410. click here to view supplementary material
