# Is Dietary Quality Associated with Depression? An Analysis of the Australian Longitudinal Study of Women’s Health Data – CORRIGENDUM

**DOI:** 10.1017/S000711452200294X

**Published:** 2023-02-14

**Authors:** Megan Lee, Joanne Bradbury, Jacqui Yoxall, Sally Sargeant

**Details of correction:** addition to acknowledgments

**Existing text:**

The authors would like to acknowledge the guidance of biostatistician Doctor Alison Bowling with the data analysis for this project; the Australian Longitudinal Study of Women’s Health for access to their data sets; the support of Professor Gita Mishra the ALSWH liaison person for our study and Doctor David Giles from the Victorian Cancer Council who constructed the DQESv2 FFQ used in this study.

This work was supported by an Australian Government Research Training Programme (RTP) stipend grant for PhD candidate Megan Lee. RTP funding is an Australian support grant for domestic or international students conducting PhD or Master of Research degrees. The project received no other grants from funding agencies, commercial or not-for-profit sectors.

This manuscript came from a PhD project. M. L. formulated the research question, designed the study, requested data through an EOI from the ALSWH, cleaned and analysed the data with contribution from A. B. and J. B., interpreted the findings and wrote the article under the supervision of S. S., J. Y. and J. B.

The authors acknowledge no conflicts of interest for this research project.

**Corrected text should read:**

The research on which this paper is based was conducted as part of the Australian Longitudinal Study on Women’s Health by the University of Queensland and the University of Newcastle. We are grateful to the Australian Government Department of Health and Aged Care for funding and to the women who provided the survey data.

The authors would like to acknowledge the guidance of biostatistician Doctor Alison Bowling with the data analysis for this project; the Australian Longitudinal Study of Women’s Health for access to their data sets; the support of Professor Gita Mishra the ALSWH liaison person for our study and Professor Graham Giles and Professor Roger Milne of the Cancer Epidemiology Centre of Cancer Council Victoria, for permission to use the Dietary Questionnaire for Epidemiological Studies (Version 2), Melbourne: Cancer Council Victoria, 1996

This work was supported by an Australian Government Research Training Programme (RTP) stipend grant for PhD candidate Megan Lee. RTP funding is an Australian support grant for domestic or international students conducting PhD or Master of Research degrees. The project received no other grants from funding agencies, commercial or not-for-profit sectors.

This manuscript came from a PhD project. M. L. formulated the research question, designed the study, requested data through an EOI from the ALSWH, cleaned and analysed the data with contribution from A. B. and J. B., interpreted the findings and wrote the article under the supervision of S. S., J. Y. and J. B.

The authors acknowledge no conflicts of interest for this research project.

**Details of correction:** changes to paper title

**Existing text:** Is dietary quality associated with depression? An analysis of the Australian longitudinal study of women’s health data

**Corrected text should read:** Is dietary quality associated with depression? An analysis of the Australian Longitudinal Study on Women’s Health data

