# Supplementary material for: Is dietary quality associated with depression? An analysis of the Australian Longitudinal Study on Women’s Health data
Source: Br J Nutr. 2022 Jul 27;129(8):1380–7. doi: 10.1017/S0007114522002410 (PMC10024974; doi:10.1017/S0007114522002410)
Supplement: Supplementary file 1 [file S0007114522002410sup001.docx]

**Supplementary Table**

*Hierarchical Linear Mixed-Effects Model of ARFS Total Score on CESD-10^a^ (Unstandardised Beta Coefficients)*

|  | Step1 | Step 2 | Step 3 | Step 4 | Step 5 | Step 6 | Step 7 |
| --- | --- | --- | --- | --- | --- | --- | --- |
| ARFS^b^ total score | -0.06^**^ | -0.05^**^ | -0.05^**^ | -0.04^**^ | -0.04^**^ | -0.03^**^ | -0.03^**^ |
| Year |  |  |  |  |  |  |  |
| 2003 |  |  |  |  |  |  |  |
| 2009 | -0.23^**^ | -0.38^**^ | -0.55^**^ | -0.26^**^ | -0.27^**^ | -0.23^**^ | -0.21^**^ |
| Anxiety |  | 3.91^**^ | 3.85^**^ | 1.90^**^ | 1.88^**^ | 1.82^**^ | 1.81^**^ |
| BMI^c^ |  |  | 0.12^**^ | 0.09^**^ | 0.09^**^ | 0.08^**^ | 0.08^**^ |
| Social function |  |  |  | -0.13^**^ | -0.13^**^ | -0.13^**^ | -0.13^**^ |
| Alcohol status |  |  |  |  |  |  |  |
| Non-drinker |  |  |  |  |  |  |  |
| Low-risk drinker |  |  |  |  | -0.03 | -0.08 | -0.04 |
| Rarely drinks |  |  |  |  | 0.27^*^ | 0.24 | 0.24 |
| Risky drinker |  |  |  |  | 0.84^**^ | 0.62^**^ | 0.67^**^ |
| High-risk drinker |  |  |  |  | 1.66^**^ | 1.32^*^ | 1.33^*^ |
| Smoking status |  |  |  |  |  |  |  |
| Never smoked |  |  |  |  |  |  |  |
| Ex-smoker |  |  |  |  |  | 0.31^**^ | 0.26^*^ |
| Smokes <10 day |  |  |  |  |  | 0.36^*^ | 0.31^*^ |
| Smokes 10-19 day |  |  |  |  |  | 0.91^**^ | 0.80^**^ |
| Smokes >= 20 day |  |  |  |  |  | 1.11^**^ | 0.98^**^ |
| Education |  |  |  |  |  |  |  |
| No qualification |  |  |  |  |  |  |  |
| Year 10 |  |  |  |  |  |  | -0.49 |
| Year 12 |  |  |  |  |  |  | -0.57 |
| Trade certificate |  |  |  |  |  |  | -0.72 |
| Diploma |  |  |  |  |  |  | -0.65 |
| Undergraduate |  |  |  |  |  |  | -0.95^*^ |
| Postgraduate |  |  |  |  |  |  | -0.89^*^ |
| N | 14,791 | 14,791 | 14,791 | 14,791 | 14,791 | 14,791 | 14,791 |
| AIC^d^ | 89,862.46 | 89,042.38 | 88,818.91 | 83,353.91 | 83,315.68 | 83,257.67 | 83,246.28 |
| Logs-ratio test |  | 642.09^**^ | 225.46^**^ | 5,466.94^**^ | 46.30^**^ | 66.00^**^ | 23.39^**^ |

*Note.* * p < .05 ** p < .001 ^a^CESD-10, Centre for Epidemiological Studies Depression Score. ^b^ARFS, Australian Recommended Food Score. ^c^BMI, Body Mass Index. ^d^ AIC, Akaike Information Criterion
